# The influence of fasting on energy and nutrient intake and their corresponding food sources among 6-23 months old children in rural communities with high burden of stunting from Northern Ethiopia

**DOI:** 10.1186/s12937-022-00759-z

**Published:** 2022-01-14

**Authors:** Mekonnen Haileselassie, Getachew Redae, Gebretsadik Berhe, Carol J. Henry, Michael T. Nickerson, Afework Mulugeta

**Affiliations:** 1 School of Public Health, College of Health Sciences, Mekelle University, Mekelle, Ethiopia; 2 Tigray National Regional State, Bureau of Science and Technology, Mekelle, Tigray Ethiopia; 3 College of Pharmacy and Nutrition, University of Saskatchewan, Saskatoon, Canada; 4 Department of Food and Bioproduct Sciences, University of Saskatchewan, Saskatoon, Canada

**Keywords:** Dietary intake, Children, Orthodox fasting

## Abstract

**Background:**

Limited studies in Ethiopia showed that infants and young children are at high risk of inadequate intake of energy and nutrients. However, inclusive assessment of both nutrient intakes and their food sources are lacking. We aimed at assessing energy and nutrient intakes and their food sources during religious fasting and non-fasting periods among 6–23 months old children in Northern Ethiopia.

**Methods:**

Data for this longitudinal study were collected following repeated multiple-pass 24-h dietary recall technique through face-to-face interviews with primary caregivers. Using a two-stage systematic random sampling method, a total of 570 and 551 children participated respectively in the lent fasting and non-fasting periods. Energy and nutrient intakes were estimated and compared with WHO daily requirements. All foods that a child consumed on the day preceding the date of data collection were recorded and processed with database software. Chi-square and t- tests were used to analyze the data. Non-normally distributed data were analyzed using Wilcoxon signed-rank test and statistical significance was set at *p* < 0.05.

**Results:**

The overall prevalence of child stunting was 41.4%. Almost all of children (99.6%) consumed grains, roots, and tubers. The inadequacy prevalence of energy, protein and eight selected micronutrients (calcium, iron, zinc, vitamin A, thiamin, riboflavin, niacin, vitamin C) intake were 96.2, 44.9, and 95.5%, respectively. Calcium and zinc were the highest (100%) deficits observed across all age groups. Although consumption of animal source foods (ASFs) was very low (dairy 10.1%, meat 2.3% and eggs 23.6%), there was significantly higher consumption of meat and eggs during the non-fasting compared to fasting period (*p* < 0.001).

**Conclusions:**

Inadequate intake of energy and nutrients was common among 6–23 months old children. Cereals were found to be the main sources of many of the nutrients. The consumption of ASFs among 6–23-month-old children was low which was also affected by the religious fasting period. Hence, strengthening social and behavior change communication, supporting rural households to raise poultry and small ruminants is recommended.

## Introduction

Malnutrition is an underlying cause for child mortality, particularly among low socioeconomic communities in developing countries [1]. It has severe short-and long-term health consequences including child growth restriction, poor cognition and educational performance, lost productivity and increased risk of nutrition-related chronic diseases [2]. Globally, 144 and 47 million children under- five suffer from stunting and wasting respectively [3].

Despite the remarkable achievements in improving children’s nutrition over the past two decades, child malnutrition is still a major public health challenge in Ethiopia. The national prevalence of stunting, underweight and wasting among children under five was 36.8, 21.1 and 7.2% in 2019, respectively [4]. Similarly, 57% of children under five were anemic, only 14% had an adequately diverse diet, and just 7% of children aged 6–23 months meet the minimum acceptable dietary standards [5].

The current level of child malnutrition may persist if the socio-cultural and religious determinants to child feeding practices are not thoroughly investigated and interventions are not designed to mitigate their effects on child nutrition. The Orthodox Tewahedo Christians is among the religions with 43.5% adherents in Ethiopia [6]. During the Orthodox fasting period, eating animal source foods (ASFs) such as flesh foods, eggs and dairy products are strictly forbidden except for children and pregnant women [7]. There are more than 150 days per year that most adults do not consume ASFs during the Orthodox fasting period [8, 9]. Although fasting have positive health-related markers among adults [10, 11], the practices of fasting by adult caregivers/parents may affect the type of complementary foods to be offered for children. During this period, though children are exempted from fasting, ASFs may not be prepared at home and be available for them due to mother/caregivers fear of contaminating utensils during cooking family foods [9, 12, 13]. Therefore, children miss ASFs which are energy and nutrient-dense, high quality and easily digestible protein sources and an efficient and easily absorbable source of micronutrients like iron, zinc, vitamin A, vitamin B12, riboflavin and calcium [14]. Hence, 6–23 months of age children are at high risk for energy and micronutrient deficiencies [15] because they have high needs to energy and micronutrient intakes [16]. To date, the few studies in Ethiopia [17–19] described the intake of energy and some selected nutrients among children under five. However, none of them have taken into account the intake of many essential nutrients and their food sources in areas where chronic malnutrition is high.

Therefore, this study was conducted to investigate the influence of fasting on energy and nutrient intakes and their corresponding food sources among 6–23 months old children from the rural communities in Northern Ethiopia where the burden of child stunting is unacceptably high. This will enable program managers to design interventions to improve intake and quality of complementary foods for 6–23 months old children.

## Materials and methods

### Study area, sample size and sampling techniques

The study was conducted in the rural areas of Samre Seharti and Tanqua Abergele, both food insecure districts of Tigray Region, Northern Ethiopia, with a total population of 144,527 and 120,180, respectively [20]. Both are intervention districts of the Seqota Declaration, with a target of achieving an end to childhood stunting by 2030 [21].

A single population proportion formula, *n* = Z^2^_1- α/2_ *P* (1-*P*) /d^2^ was used to determine the sample size and was estimated to be 366. The following parameters were taken into consideration during the sample size calculation: 95% Confidence level, 5% desired degree of precision and 39.3% stunting prevalence of Tigray region [5], 1.5 design effect (to account for the heterogeneity between Tabias of the district about the measured indicator) and a 10% non-responses rate. Thus the final sample size was computed as 604 children whose mothers had been practicing fasting during the religious fasting period.

**Fig. 1:**
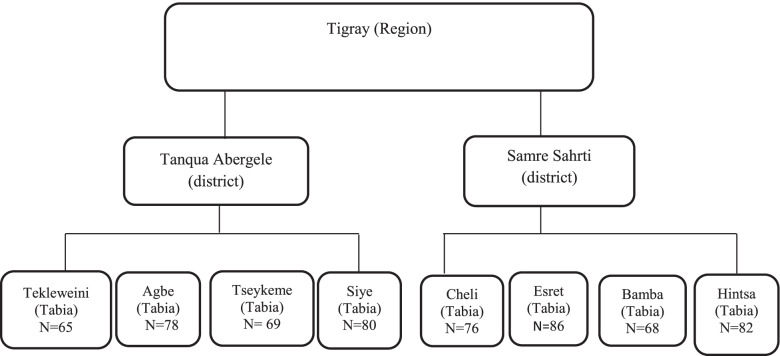
Schematic representations of the sampling technique for the quantitative study in Tigray Region, Northern Ethiopia, 2019

Eight Tabias were selected from the forty-three Tabias in the two districts of Samre Seharti and Tanqua Abergele, using simple random sampling technique. The sample size was proportionately divided into the randomly eight selected Tabias. Figure 1 depicts the selected tabias and the sampled households. Study participants in each nominated Tabia were selected by systematic random sampling method.

### Inclusion and exclusion criteria

Children with 6–23 months age, apparently healthy and breastfeeding during the study periods were included. Breastfeeding children were selected due to the reason that continued breastfeeding rates at two years were high (92.4%) at the regional level [5]. Non-fasting mothers, those with twins and not permanent dwellers (mothers have no plans to stay longer than six months) were excluded from the study.

### Study design, duration and participants

A longitudinal study design was carried out in both lent fasting and non-fasting periods. Mothers with 6–23 months old children were answer as indirect responders. The first and second round study was conducted from March 21–April 17/2019 and May 23–June 15/2019 respectively.

### Data collection

#### Socio-demographic characteristics and anthropometry

Information on socio-demographic characteristics of the study participants such as children’s age and sex, family size of households, parents’ educational level were collected using a structured questionnaire. Height (recumbent length) was measured using a portable child length measuring wooden board (Shorr board, California) to the nearest 0.1 cm. While weight of the child was measured using a calibrated high precision portable digital scale measured to the nearest 0.1 kg [17, 22]. Data were collected by health professionals who were well trained and skilled in child anthropometric measurements. Duplicate measurements (length and weight) were taken following standard procedures [23]. Height-for-age, weight-for-age, and weight-for-height were calculated according to the WHO Standard Reference [24] using the software ENA 2015. The nutritional status indicators namely stunting, underweight, and wasting were defined as Length-for-age z-score (LAZ), Weight-for-age z-score (WAZ), and Weight-for-length z-score (WLZ) < − 2 Z-scores, respectively.

### Dietary diversity

An interactive 24-h dietary recall method was used to assess dietary diversity in each household by asking the child’s primary caregiver about breastfeeding and all foods consumed by each child on the previous day [25]. Food items consumed were classified into either of seven food groups: (1) cereals; (2) meat (beef, lamb, goat, chicken, liver, kidney, heart or other organs and fish); (3) milk and milk products; (4) eggs; (5) legume and pulses; (6) Vitamin A-rich fruits and vegetables; and (7) Other fruits and vegetables [17]. Then children’s minimum meal frequency (MMF), and minimum diet diversity (MDD) were computed using the 24-h recall data. Children receiving four or more food groups were categorized as meeting the minimum dietary diversity; or else, they were considered as getting low minimum dietary diversity [25].

### Dietary intakes and food sources

An interactive two round 24-h dietary recall was conducted with the primary caregivers using multiple pass technique validated for use in developing countries [26]. The technique included a quick list of all consumed foods and drinks, time, place, ingredients, detailed description of listed food items, preparation and cooking method, and review of the recall. In some cases, the sibling and father of the child assisted the primary caregiver to recall the food intake of the child. The recruited data collectors had experience with similar data collection techniques and prior to collection of data; they were trained and conducted a pilot test to further modify the questionnaire. The portion sizes of each food/drink was estimated using local measurement aids such as spoons, feeding bottle cups, ladles and bowls. Digital food photos were also used as a memory aid and to estimate the portion sizes of foods given by primary caregivers [17]. Data collectors had also salted replica of common local infant and young child foods in the study area, such as wheat (*Triticum aestivum*) and sorghum (*Sorghum bicolour*) bread, enjera, porridge, gruel, shallot, tomato, and stew. To estimate portion size, each participant was asked to put the amount of food that is equivalent to the food actually eaten if actual food is available at their home or salted replica on a food weighing scale in grams (calibrated portable electronic kitchen scale, Yongkang Zhezhong Weighing Apparatus Factory, China). The respondents were also asked to estimate the portion of leftover. Every respondent was also asked for snack, fluid and outdoor consumption of food after they completed the recall. All days of the week were equally represented in the final sample. All the collected food items were grouped into the seven food groups by the researchers. Food items that contributed 10% and above to the intakes of energy and nutrients were presented by the children’s age group (6–8 months, 9–11 months and 12–23 months) between the lent fasting and non-fasting periods.

### Estimation of nutrient intakes and nutrient density

Energy and nutrient estimation of diets, recipes, or commercial food items was conducted using the Ethiopian food composition table [27]. Missing values were completed using the USDA nutrient database [28]. The median energy and nutrient intakes from complementary foods were calculated for fasting and non-fasting periods separately for each child’s age group. Energy and nutrient intake of breastfed children were compared to estimated needs from complementary foods assuming average intake of breast milk and composition [29, 30]. The “nutrient density per 100kcal” of the diet was calculated by dividing nutrient intake by the total energy intake (in kcal) as described in Dewey and Brown [29]. The nutrient densities were compared with estimated needs for energy and selected nutrients that would allow children to meet their daily nutrient requirements based on WHO/FAO recommended intakes [31].

### Data quality control

In the beginning prior to data collection, data collectors were trained and conducted a pilot test to further modify the questionnaire. A strong follow-up and continuous support were also given for them while they were in the field. The nutrient content of children’s diet was assessed using the Ethiopian food composition table [27] and their feeding practices were characterized using the WHO infants and young child feeding indicators [25]. Repeated measurement across items provided consistent result and the value of Cronbach’s Alpha was 0.880 which is reliable. During the Anthropometric measurements, the scales were calibrated at the beginning of the measurement using standard weights and measuring tapes. Duplicate measurements (length and weight) were taken following standard procedures.

### Statistical analyses

Data on socio-demographic characteristics and child feeding practices were entered into Epi data version 3.1 and exported to a statistical software IBM SPSS version 20. All continuous variables were checked for normality using skewness and kurtosis test. Children’s feeding practices between fasting and non-fasting periods were compared using the t-test for continuous variables and chi-square test for categorical variables. And non-normally distributed data were analyzed using Wilcoxon signed-rank test. Statistical significance was set at *P*-values< 0.05. Multicollinearity among the variables was detected using variance inflation factor (VIF) showing that there was no multicollinearity (VIF < 10) [32].

The data about dietary intake and food sources from the dietary assessment were entered and converted into energy and nutrient intake using the NutriSurvey software program (www.nutrisurvey.de) based on Ethiopian Food Composition Tables. The dietary intakes and nutrient densities were described using medians with interquartile range due to the non-normal distributions of nutrient intakes. The adequacy of the energy and nutrient intakes were evaluated in comparison with percentage of estimated energy needs from complementary foods by calculating the energy requirements of the children based on age (in months). The percentage contribution of each food item for the energy and selected nutrients was calculated by adding the amount of a given nutrient provided by each collected food items for all individuals and dividing by the total intake of that nutrient consumed by all individuals from all food types and multiplied by 100.

## Results

### Socio-demographic and nutritional status of children

A total of 570 participants were surveyed during the Ethiopian Orthodox lent fasting periods with response rate of 93.5% and 551 in the non-fasting period “with a response rate” of 96.7%. Table 1 presents the overall socio-demographic and anthropometric characteristics of children in the study area. The overall mean age of children was 14.5 months, and 52.5% of the children were males. Mean household size of the study participants was 5.5 persons. Only 23% of the interviewed mothers had a formal education. They were all Orthodox Christian followers. About 90% of the households owned at least one type of livestock, such as cattle, sheep, goats, and chickens. The overall prevalence of child stunting, underweight, and wasting was 41.4, 21.6, and 8.6%, respectively (Table 1).

**Table 1:** Socio-demographic characteristics and nutritional status of 6–23 months old children in rural districts of Tigray Region, Ethiopia (*N* = 570)

Socio-demographic characteristics	Frequency (%)
Proportion of female children	271 (47.5)
Proportion of fathers without formal education	387 (67.9)
Proportion of mothers without formal education	439 (77)
Proportion of household livestock owners	512 (89.8)
Average family size of households (Mean ± SD)	5.5 ± 10.2
Average age of child in months (Mean ± SD)	14.5 ± 5.2
Length-for-age z-score (Mean ± SD)	−1.79 ± 1.66
Weight-for-age z-score (Mean ± SD)	−1.00 ± 1.40
Weight-for-length z-score (Mean ± SD)	−0.06 ± 1.67
Prevalence of stunting	41.4%
Prevalence of underweight	21.6%
Prevalence of wasting	8.6%

### Child dietary diversity

The overall child feeding practices between the lent fasting and non-fasting periods are summarized in Table 2. No child received meat during the lent fasting period, while only 4.5% of the children received meat during the non-fasting periods. Based on the comparisons of percent proportions, there was no significant difference (*p* > 0.05) between the lent fasting and non-fasting periods in the children’s consumption of food groups such as grains, roots and tubers, dairy products, vitamin A rich fruits and vegetables and other fruits and vegetables. However, there was a significantly higher consumption of meat and eggs and lower consumption of legumes and nuts among children during the non-fasting periods than the lent fasting periods. Minimum dietary diversity was significantly higher in the non-fasting period compared to the lent fasting periods (Table 2).

**Table 2:** Types of food groups consumed by 6–23 months age children in lent fasting and non-fasting periods in rural districts of Tigray Region, Ethiopia

Food groups	Lent Fasting Period (*n* = 570)				Non-Fasting Period (*n* = 551)				*P*-value
	6-8 m (*n* = 80)	9-11 m (*n* = 101)	12-23 m (*n* = 389)	All (*n* = 570)	6-8 m (*n* = 75)	9-11 m (*n* = 96)	12-23 m (*n* = 380)	All (*n* = 551)	
Grains, roots and Tubers	77 (96.1%)	100 (99%)	389 (100%)	566 (99.3%)	74 (98.7%)	96 (100%)	380 (100%)	550 (99.8%)	0.180
Legumes and nuts	21 (26.1%)	46 (45.5%)	269 (69.2%)	336 (58.9%)	19 (25%)	39 (40.6%)	213 (56.1%)	271 (49.2%)	< 0.0001
Dairy products	9 (11.3%)	9 (8.9%)	35 (9%)	53 (9.3%)	6 (8%)	13 (13.5%)	41 (10.8%)	60 (10.9%)	0.079
Meat	0	0	0	0	1 (1.3%)	3 (3.1%)	21 (5.5%)	25 (4.5%)	< 0.0001
Eggs	7 (8.8%)	12 (11.9%)	52 (13.4%)	71 (12.5%)	22 (29.3%)	29 (30.2%)	140 (36.8%)	191 (34.7%)	< 0.0001
Vitamin A-rich fruits and vegetables	0	0	2 (0.5%)	2 (0.4%)	0	0	4 (1.1%)	4 (0.73%)	0.415
Other fruits and vegetables	5 (6.3%)	10 (9.9%)	67 (17.2%)	82 (14.4%)	8 (10.7%)	9 (9.4%)	49 (12.9%)	66 (12%)	0.078
Minimum diet diversity	0	0	13 (3.3%)	13 (2.3%)	0	7 (7.3%)	32 (8.4%)	39 (7.1%)	< 0.0001
Minimum meal frequency	22 (27.5%)	35 (34.7%)	249 (64%)	306 (53.7%)	24 (32%)	38 (39.6%)	252 (66.3%)	314 (57%)	0.198

*m* months, *n* number of participants

### Nutrient intakes

Table 3 presents the median nutrient intake of complementary foods for the three age groups of children between the lent fasting and non-fasting periods. Except for the median intakes of vitamin A, which is the lowest for infants aged 9–11 months, all nutrients intake increased with age. Apart from protein at the age of 12–23 months, energy and the selected eight micronutrients intake from complementary foods were below the corresponding estimated needs recommended by WHO/FAO for breastfed children of 6–23 months old, in both periods. The children’s nutrient inadequacy prevalence was > 90% for energy, vitamin A, riboflavin, vitamin C and was 100% for calcium, zinc, and niacin in both lent fasting and non-fasting periods. The median intakes of all nutrients by 6–23 months old children were higher in the non-fasting periods than the lent fasting one across all age groups. Nevertheless, the median intakes of vitamin A at the age of 9–11 months children were similar in both periods.

**Table 3:** Median (IQR) daily energy and nutrient intakes of infants and young children (aged 6–23 months) from complementary foods in rural districts of Tigray Region, Ethiopia

Nutrient	Lent Fasting Period (*n* = 570)			Non-Fasting Period (*n* = 551)		
	6–8 months (*n* = 80)	9–11 months (*n* = 101)	12–23 months (*n* = 389)	6–8 months (*n* = 75)	9–11 months (*n* = 96)	12–23 months (*n* = 380)
Energy, Kcal	64.3 (24.2113.3)	142.5 (97.5, 199.0)	231 (179.8300.2)	100.3 (73.4149.4)	173.1 (133,229.7)	275.4 (204.7340.3)
Estimated need	202	307	548	202	307	548
Prev. inad	76 (95%)	99 (98%)	386 (99.2%)	70 (93.3%)	88 (91.7%)	380 (100%)
Protein, g	0.1 (0.3,1.7)	2.9 (1.6,4.5)	5.6 (3.9,7.5)	2.4 (1.4,4.1)	4.1 (2.8,6.8)	7.5 (5.5,9.7)
Estimated need	2.0	3.1	5.0	2.0	3.1	5.0
Prev. inad	66 (82.5%)	55 (54.5%)	156 (40.1%)	32 (42.7%)	28 (29.2%)	77 (20.3%)
Calcium (mg)	12 (6.7,23.6)	20 (12.8,38.1)	40.7 (24.7,64.5)	15 (8.1,35.5)	29.4 (20.9488)	44 (31,56.6)
Estimated need	211	228	346	211	228	346
Prev. inad	80 (100%)	101 (100%)	388 (99.7%)	75 (100%)	96 (100%)	380 (100%)
Iron (mg)	2 (0.9,3.0)	3.1 (1.7,4.9)	4.9 (3.2,7.6)	2.3 (1.4,3.5)	3.9 (2.8,6.9)	5.7 (3.9,8)
Estimated need	9	9.1	5.6	9	9.1	5.6
Prev. inad	80 (100%)	95 (94.1%)	223 (57.3%)	72 (96%)	85 (88.4%)	182 (47.9%)
Zinc (mg)	0.25 (0.2,0.5)	0.53 (0.37, 0.95)	1.04 (0.72,1.4)	0.4 (0.3,0.7)	0.81 (0.54,1.1)	1.23 (0.9,1.63)
Estimated need	3.3	3.4	3.7	3.3	3.4	3.7
Prev. inad	80 (100%)	101 (100%)	389 (100%)	75 (100%)	96 (100%)	380 (100%)
Vitamin A (μg RE)	(0.0,0.6)	(0.0,1.4)	0.6 (0.0,2.8)	0.4 (0.0,39.4)	(0.0,61.1)	2.04 (0.0,61.15)
Estimated need	63.0	92	126	63.0	92	126
Prev. inad	75 (93.8%)	96 (95%)	381 (97.9%)	69 (92%)	91 (94.8%)	359 (94.5%)
Thiamin (mg)	0.03 (0.01,0.06)	0.08 (0.05,0.12)	0.15 (0.1,0.2)	0.07 (0.05,0.1)	0.1 (0.07,0.17)	0.17 (0.13,0.23)
Estimated need	0.16	0.17	0.38	0.16	0.17	0.38
Prev. inad	80 (100%)	93 (92.1%)	385 (98.9%)	65 (86.7%)	73 (76%)	366 (96.3%)
Riboflavin (mg)	0.02 (0.0,0.04)	0.06 (0.03,0.09)	0.1 (0.07,0.15)	0.05 (0.05,0.07)	0.08 (0.05,0.12)	0.13 (0.09,0.17)
Estimated need	1.6	0.18	0.31	1.6	0.18	0.31
Prev. inad	80 (100%)	100 (99%)	382 (98.2%)	75 (100%)	94 (97.9%)	370 (97.4%)
Niacin (mg)	0.18 (0.14,0.4)	0.54 (0.28,0.82)	0.9 (0.49,1.4)	0.4 (0.23,0.68)	0.71 (0.37,1.14)	1.0 (0.5,1.6)
Estimated need	2.99	3.08	5.18	2.99	3.08	5.18
Prev. inad	80 (100%)	99 (98%)	382 (98.2%)	75 (100%)	87 (90.6%)	372 (97.9%)
Vitamin C, mg	0.27 (0.2,0.9)	0.82 (0.31,1.73)	1.46 (0.5,3.1)	0.98 (0.36,1.54)	1.5 (0.45,2.6)	1.9 (0.6,3.7)
Estimated need	3.0	5.4	8.0	3.0	5.4	8.0
Prev. inad	77 (96.3%)	101 (100%)	387 (99.5%)	73 (97.3%)	96 (100%)	380 (100%)

*RE* retinol equivalent, *Prev* inad, Prevalence of inadequacy; iron and zinc were considered as moderate bioavailable

### Nutrient densities

The median nutrient densities of protein for children of all age groups in the lent fasting and non-fasting periods exceed desired protein density from complimentary foods. Similarly, iron for 12–23 months (in both periods) and thiamin for 9–23 months (non-fasting periods) was found higher than the corresponding estimated needs where moderate bioavailability was assumed for iron and zinc. However, the rest of micronutrients from complementary foods across all age groups were below the desired density in both periods. The highest deficits were observed for the nutrient density of calcium across all age groups of children during the lent fasting and non-fasting periods (Table 4).

**Table 4:** Median (first, third quartile) nutrient densities of complementary foods in relation to estimated needs among children aged 6–23 months in rural districts of Tigray Region, Ethiopia

Nutrient density (/100 kcal)	Lent Fasting Period (*n* = 570)			Non-Fasting Period (*n* = 551)		
	6–8 m (*n* = 80)	9–11 m (*n* = 101)	12–23 m (*n* = 389)	6–8 m (*n* = 75)	9–11 m (*n* = 96)	12–23 m (*n* = 380)
Protein, g	1.3 (1.2,2.2)	2.1 (1.5,2.6)	2.3 (2.0,2.7)	2.2 (1.6,3.3)	2.3 (1.9,3.0)	2.6 (2.2,3.3)
Estimated need	1.0	1.0	0.9	1.0	1.0	0.9
Calcium (mg)	21.3 (13.1,30.1)	16.5 (10.6,27)	16.7 (10.2,26.6)	16.2 (9.9,23.5)	18 (11.8,27)	16.2 (11.0,23.3)
Estimated need	105	74	63	105	74	63
Iron (mg)	3.4 (1.6,8.1)	2.1 (1.4,4.3)	2.0 (1.5,3.1)	2.1 (1.5,3.2)	2.3 (1.7,3.3)	2.0 (1.6,2.8)
Estimated need	4.5	3.0	1.0	4.5	3.0	1.0
Zinc (mg)	0.5 (0.34,0.97)	0.5 (0.3,0.7)	0.5 (0.4,0.6)	0.5 (0.4,0.6)	0.5 (0.4,0.6)	0.5 (0.4,0.6)
Estimated need	1.6	1.1	0.6	1.6	1.1	0.6
Vitamin A (mg RE)	(0, 1.14)	(0.0,1.0)	0.3 (0.0,1.2)	0.4 (0.0,31.2)	0.4 (0.0,24.8)	0.8 (0.0,24.6)
Estimated need	31	30	23	31	30	23
Thiamin mg	0.05 (0.04,0.07)	0.06 (0.05,0.08)	0.06 (0.05,0.08)	0.07 (0.06,0.09)	0.07 (0.05,0.08)	0.07 (0.05,0.08)
Estimated need	0.08	0.06	0.07	0.08	0.06	0.07
Riboflavin mg	0.03 (0.0,0.06)	0.04 (0.03,0.06)	0.05 (0.03,0.06)	0.05 (0.04,0.06)	0.05 (0.04,0.06)	0.05 (0.04,0.06)
Estimated need	0.08	0.06	0.06	0.08	0.06	0.06
Niacin mg	0.5 (0.35,0.73)	0.4 (0.3,0.6)	0.4 (0.2,0.6)	0.5 (0.3,0.6)	0.4 (0.3,0.6)	0.4 (0.2,0.6)
Estimated need	1.5	1.0	0.9	1.5	1.0	0.9
Vitamin C, mg	1.1 (0.29,1.13)	0.7 (0.3,1.1)	0.7 (0.2,1.2)	0.9 (0.4,1.5)	0.8 (0.3,1.4)	0.8 (0.2,1.3)
Estimated need	1.5	1.8	1.5	1.5	1.8	1.5

*m* months, *n* number of participants

### Contribution of food items to children’s energy and nutrient intakes

Contribution of food types for nutrient intakes of 6–23 month old children in lent fasting and non-fasting periods is described (Table 5). Although more than 100 food types were collected from the study area, only nine food items contributed 10% and above to children’s energy and nutrient intakes. Cereals, mainly sorghum remained the most consumed food type by the children in both lent fasting and non-fasting periods. Sesame and linseed were also good sources of calcium during the lent fasting periods of the children’s diet.

**Table 5:** Contribution of food items (at least 10%) to nutrient intakes of 6–23 month old children in lent fasting and non-fasting periods in rural districts of Tigray Region, Ethiopia

Nutrient	Food groups	Lent Fasting Period (*n* = 570)			Non-Fasting Period (*n* = 551)		
		6–8 months	9–11 months	12–23 months	6–8 months	9–11 months	12–23 months
Energy, Kcal Energy, Kcal	Cereals	Sorghum: mixed, red and white, (porridge & injera) (15%)	Sorghum: mixed, red and white, (porridge, bread & enjera) (25.3%)	Sorghum: mixed, red and white, (porridge, bread & enjera) (28.3%)	Sorghum: mixed, red and white, (porridge, bread & enjera) (30.3%)	Sorghum: mixed, red and white, (porridge, bread & enjera) (35.9%)	Sorghum: mixed, red and white, (porridge, bread & enjera) (33.6%)
		Tef: mixed, whit & red, (gruel & porridge) (29%)	Tef: mixed & red, (gruel, bread, enjera & porridge) (15.7%)	Sorghum (60%) + tef (40%) enjera (11.3%)	Wheat: mixed Bread, enjera & Porridge (10.4%)	–	Wheat: mixed (Bread, enjera & Porridge) (10.7%)
		–	–	Com (maize): white & yellow (porridge, bread, gruel & enjera) (11.9%)	–	Com (maize): yellow & white (gruel, bread, enjera & Porridge) (10.3%)	–
	Eggs	–	–	–	Egg (Boiled & fried) (10.3%)	Egg (Boiled & fried) (10.9%)	Egg (Boiled & fried) (13.7%)
Protein, g	Cereals	Sorghum: mixed, red and white, (porridge & injera) (17.7%)	Sorghum: mixed, red and white, (porridge, bread & enjera) (20.5%)	Sorghum: mixed, red and white, (porridge, bread & enjera) (22.7%)	Sorghum: mixed, red and white, (porridge, bread & enjera) (20%)	Sorghum: mixed, red and white, (porridge, bread & enjera) (25.7%)	Sorghum: mixed, red and white, (porridge, bread & enjera) (23.6%)
		Tef: mixed, white, red, (gruel & porridge) (23.2%)	Tef: mixed & red, (gruel, bread, enjera & porridge) (18.4%)	Wheat: mixed (Bread, enjera & Porridge) (10.1%)	–	–	–
		–	–	Sorghum (60%) + tef (40%) enjera (14.2%)	–	–	–
		–	–	Com (maize). *Zea mays* L.: white & yellow (porridge, bread, gruel & enjera) (10.5%)	–	–	–
	Eggs	–	Egg (Boiled & fried) (12.7%)	Egg (Boiled & fried) (10.7%)	Egg (Boiled & fried) (22%)	Egg (Boiled & fried) (29.3%)	Egg (Boiled & fried) (25.2%)
Calcium, mg	Cereals	Sorghum: mixed, red and white, (porridge & injera) (11.1%)	Sorghum: mixed, red and white, (porridge, bread & enjera) (14.6%)	Sorghum: mixed, red and white, (porridge, bread & enjera) (13%)	Sorghum: mixed, red and white, (porridge, bread & enjera) (12.8%)	Sorghum: mixed, red and white, (porridge, bread & enjera) (19.8%)	Sorghum: mixed, red and white, (porridge, bread & enjera) (17.3%)
		Tef: mixed, white, red, (gruel & porridge) (33.4%)	Tef: mixed & red, (gruel, bread, enjera & porridge) (21.2%)	Tef: mixed, white & red, (gruel, bread, enjera & porridge) (11.7%)	Tef: mixed & red, (gruel, enjera & porridge) (12.4%)	Tef: mixed & red, (gruel, enjera & porridge) (12.6%)	Sorghum (60%) + tef (40%) enjera (17.5%)
		–	Sorghum (60%) + tef (40%): (enjera) (12%)	Sorghum (60%) + tef (40%) (22.8%)	–	Sorghum (60%) + tef (40%): (enjera) (10%)	–
	Legumes & nuts	–	Sesame and Linseed: (roasted & dried) (10.2%)	Sesame and Linseed: (roasted & dried) (14.5%)	–	–	–
	Eggs	–	–	–	Egg (Boiled & fried) (18%)	Egg (Boiled & fried) (18.8%)	Egg (Boiled & fried) (19.2%)
	Dairy products	Milk (boiled) (27.4%)	Milk (boiled & Whey) (16%)	Milk (boiled & Whey) (10.6%)	Milk (boiled) (26.1%)	Milk (boiled & Whey) (14.9%)	Milk (boiled & Whey) (11%)
Iron, mg	Cereals	Sorghum: mixed, red and white, (porridge & injera) (10%)	Sorghum: mixed, red and white, (porridge, bread & enjera) (16.5%)	Sorghum: mixed, red and white, (porridge, bread & enjera) (19.5%)	Sorghum: mixed, red and white, (porridge, bread & enjera) (18.9%)	Sorghum: mixed, red and white, (porridge, bread & enjera) (21.2%)	Sorghum: mixed, red and white, (porridge, bread & enjera) (22.5%)
		Tef: mixed, white, red, (gruel & porridge) (74.5%)	Tef: mixed & red, (gruel, bread, enjera & porridge) (43.7%)	Tef: mixed, white & red, (gruel, bread, enjera & porridge) (28.6%)	Tef: mixed & red, (gruel, enjera & porridge) (29.3%)	Tef: mixed & red, (gruel, bread, enjera & porridge) (24.6%)	Tef: mixed, white & red, (gruel, enjera & porridge) (15.4%)
		–	Sorghum (75%) + maize (25%): (enjera) (12.9%)	–	Sorghum (50%) + maize (50%) (enjera & bread) (15.2%)	Sorghum (75%) + maize (25%): (enjera & unleavened bread) (23.8%)	Sorghum (50%) + maize (50%) (enjera & bread) (18.4%)
	Eggs	–	–	–	Egg (Boiled & fried) (10.6%)	–	–
Zinc, mg	Cereals	–	Sorghum: mixed, red and white, (porridge, bread & enjera) (18.1%)	Sorghum: mixed, red and white, (porridge, bread & enjera) (21.4%)	Sorghum: mixed, red and white, (porridge, bread & enjera) (19.7%)	Sorghum: mixed, red and white, (porridge, bread & enjera) (25.1%)	Sorghum: mixed, red and white, (porridge, bread & enjera) (26.1%)
		Tef: mixed, white, red, (gruel & porridge) (63.1%)	Tef: mixed & red, (gruel, bread, enjera & porridge) (30.9%)	Tef: mixed, white & red, (gruel, bread, enjera & porridge) (19.1%)	Tef: mixed & red, (gruel, enjera & porridge) (20.8%)	Tef: mixed & red, (gruel, bread, enjera & porridge) (19.3%)	–
		–	Com (maize): white & yellow (porridge, bread, gruel & enjera) (12.2%)	Com (maize): white & yellow (porridge, bread, gruel & enjera) (12.8%)	–	Com (maize): yellow & white (gruel, bread, enjera & porridge) (11.4%)	–
	Eggs	–	–	–	Egg (Boiled & fried) (19.4%)	Egg (Boiled & fried) (17.1%)	Egg (Boiled & fried) (16.1%)
Vitamin A, μg	Eggs	Egg (Boiled & fried) (26.7%)	Egg (Boiled & fried) (65.57%)	Egg (Boiled & fried) (80.5%)	Egg (Boiled & fried) (64.9%)	Egg (Boiled & fried) (96%)	Egg (Boiled & fried) (92.1%)
	Fruits	Mango: fresh (31.9%)	–	–	Mango: fresh (10.5%)	–	–
Thiamin, mg	Cereals	Sorghum: mixed, red & White (porridge, injera & bread) (41.7%)	Sorghum: mixed, red and white, (porridge, bread & enjera) (32.3%)	Sorghum: mixed, red and white, (porridge, bread & enjera) (37.8%)	Sorghum: mixed, red and white, (porridge, bread & enjera) (44.2)	Sorghum: mixed, red and white, (porridge, bread & enjera) (46.2%)	Sorghum: mixed, red and white, (porridge, bread & enjera) (38.8%)
		Tef: white & mixed (porridge) (25%)	–	Tef: mixed & red, (gruel, bread, enjera & porridge) (17.7%)	–	–	Wheat (50%) + maize (50%): (thick enjera, unleavened bread) (10.3%)
		Wheat: mixed & white (bread & (porridge) (16.7%)	Wheat: mixed (Bread, enjera & Porridge) (14.5%)	Wheat: mixed (Bread, enjera & Porridge) (16.4%)	Wheat: mixed (Bread, enjera & Porridge) 23.3%)	Wheat: mixed (Bread & Porridge) (9.9%)	Wheat: mixed (Bread, enjera & Porridge) (10.3%)
		–	Com (maize): white & yellow (porridge, bread, gruel & enjera) (16.1%)	Com (maize): white & yellow (porridge, bread & enjera) (11.6%)	–	–	–
	Eggs	–	–	–	Egg (Boiled & fried) (16.3%)	Egg (Boiled & fried) (25.3%)	Egg (Boiled & fried) (21.1%)
Riboflavin, mg	Cereals	Sorghum: mixed. (porridge & injera) (25%)	Sorghum: mixed, red and white, (porridge, bread & enjera) (31.6%)	Sorghum: mixed, red and white, (porridge, bread & enjera) (31.7%)	Sorghum: mixed, red and white, (porridge, bread & enjera) (41.7%)	Sorghum: mixed, red and white, (porridge, bread & enjera) (51.1%)	Sorghum: mixed, red and white, (porridge, bread & enjera) (36.7%)
		–	Tef: mixed, white & red, (bread, enjera & porridge) (29%)	Tef: mixed, white & red, (bread, enjera & porridge) (20%)	–	–	Tef: mixed, white & red, (bread, enjera & porridge) (10.9%)
		–	–	Wheat: mixed (Bread, enjera & Porridge) (15.8%)	–	Wheat: mixed (Bread, enjera & Porridge) (16.9%)	Wheat: mixed (Bread, enjera & Porridge) (15%)
	Eggs	Egg (fried) (12.5%)	Egg (Boiled & fried) (16.7%)	Egg (Boiled & fried) (10.5%)	Egg (Boiled & fried) (22.2%)	–	Egg (Boiled & fried) (15.7%)
	Dairy products	Milk (boiled) (50%)	–	–	Milk (boiled) (33.3%)	–	–
Niacin, mg	Cereals	Sorghum: mixed, red & White (porridge, injera & bread) (28.6%)	Sorghum: mixed, red and white, (porridge, bread & enjera) (52.5%)	Sorghum: mixed, red and white, (porridge, bread & enjera) (48.5%)	Sorghum: mixed, red and white, (porridge, bread & enjera) (43.4)	Sorghum: mixed, red and white, (porridge, bread & enjera) (58.6%)	Sorghum: mixed, red and white, (porridge, bread & enjera) (58.4%)
		Tef: mixed, white & red (gruel & porridge) (47.2%)	Tef: mixed, white & red, (gruel, bread, enjera & porridge) (19.8%)	Tef: mixed, white & red, (gruel, bread, enjera & porridge) (12.6%)	Tef: mixed & red, (gruel, enjera & porridge) (15%)	Tef: mixed, white& red, (gruel, enjera & porridge) (12.5%)	–
		–	–	Wheat: mixed (Bread, enjera & Porridge) (11.2%)	Wheat: (mixed & red) (Bread, enjera & Porridge) (12.6%)	–	–
Vitamin C, mg	Cereals	Sorghum: mixed, red & White (porridge, injera & bread) (52%)	Sorghum: mixed, red and white, (porridge, bread & enjera) (64.6%)	Sorghum: mixed, red and white, (porridge, bread & enjera) (65.4%)	Sorghum: mixed, red and white, (porridge, bread & enjera) (53.1%)	Sorghum: mixed, red and white, (porridge, bread & enjera) (74.5%)	Sorghum: mixed, red and white, (porridge, bread & enjera) (73.3%)
		Tef: mixed, white & red (gruel) (11.3%)	–	–	–	–	–
	Fruits	Mango fresh (30.7%)	–	–	Mango fresh (22.3%)	–	–

Eggs provided the major sources of vitamin A, in all age groups of the children in both fasting and non-fasting periods. The mixed, red and white sorghum was the highest source of all B- vitamins (thiamin, riboflavin, and niacin), and vitamin C across all ages of the study children during the lent fasting and non-fasting periods.

## Discussion

The aim of the study was to assess energy intakes and dietary nutrient adequacy and their food sources during lent fasting and non-fasting periods among rural 6–23 months old children in Northern Ethiopia. In general, our findings indicated that children’s complementary diets were poor both in quantity and quality. The total intake of energy, protein and eight selected micronutrients by infants and young children were not in accordance to WHO/FAO [31] recommendations. Shortfalls of energy and nutrients were observed. This large shortfall for the diet of the study children may be explained, in part by the highest prevalence of child stunting (41.4%) in the study area.

### Feeding practices

Our study showed that cereals were the most ubiquitous along with legumes and nuts claimed to be fed by the children frequently. Only 7.1 and 2.3% of the infants and young children met the minimum dietary diversity of four or above food groups per day during the non-fasting period and the lent fasting period, respectively. Our finding was much lower than other studies from Ethiopia 21.8% [33], 59.9% [34], 38% [35], 27.3% [36], and (11.3%) [37]. This could be associated with poor feeding habit, low economic status, poor nutrition knowledge, social norms, and beliefs among the study communities [13, 33, 37]. Thus, social and behavioral change communication and health interventions suggested to improve the feeding practices and stunting among infants and young children [13, 38].

Although 90% of household own at least one type of livestock; we have found that the consumption of a diet composed of meat was 4.5% in non-fasting period and (0.0%) during the lent fasting periods. These results are similar with a previous study in North West Ethiopia (0.0%) [9] and in Southern Ethiopia (1.6%) [39]. This is further supported by previous evidence that households who owned some livestock, such as chickens, goats, and cows mainly used them as an income-generating activity rather than for home consumption [19]. Thus, implementing appropriate nutrition education and improving the socioeconomic status of the community could advance the dietary diversity of infants and young children during the fasting and non-fasting periods [19, 40].

### Inadequacy of nutrient intakes and micronutrient densities

The present study depicted that the average inadequacy of nutrients intake was particularly higher for energy (96.2%) and eight selected micronutrients (95.5%) compared to protein (44.9%) in both lent fasting and non-fasting periods. Although low adequacy intake has been reported from earlier studies in Ethiopia [17, 19] and Bangladesh [41], the severity of nutrient intake inadequacy was higher in our study children. This large shortfall in the diet of the study children could not be adequate for catch-up growth trajectory of the infant and young children in the study area.

It is evidenced that micronutrients are crucial for chemical processes that assure the survival, growth, and functioning of vital human systems [42]; yet, our study indicated that children who had received adequate intakes of energy and eight selected micronutrients was (1.6 and 2.4%, respectively) in lent fasting period and (4.9 and 7.3%, respectively) in non-fasting period. This could be due in part to inappropriate feeding practices [43] and low energy and nutrient density of complementary foods [17, 44, 45].

Complementary foods should contain ASFs or be fortified in some way especially for nutrients that must come from ASFs such as iron (97–98%) and zinc (80–87%) [29]. However, the consumption of ASFs, mainly meat, by children in the study communities was lacking during the lent fasting period and low during the non-fasting period. The micronutrient intakes of these children were unlikely to achieve their estimated need because of the very low micronutrient densities of their complementary diets for all nutrients. Although the consumption of meat and eggs during the non-fasting period was higher than the lent fasting one, their contribution to nutrient intakes among the study children was inadequate at both periods, which did not allow time (fasting/non-fasting) variation in nutrient intakes to be considered. This is because the consumption level of different food groups in both periods was inadequate among infants and young children.

Although all of the nutrients received by the children were below the desired needs in lent fasting and non-fasting periods of all age groups, children were able to achieve better adequacy for protein and iron as compared to other nutrients. Indeed, similar to our findings, earlier studies reported that protein and iron nutrient densities met the desired needs of children [17, 46]. Others reported 100% inadequacy of nutrient intake for calcium and zinc across all age groups of the children during both periods of lent fasting and non-fasting. This may arise either from low or absence of foods rich calcium and zinc in the diets of children, which are mainly found in ASFs. Similar to the current findings, the highest deficiency of calcium was identified in the diets of infants and young children elsewhere in Ethiopia [19] and Bangladesh [41]. Hence, extension services should specifically focus on educating women to own livestock for household consumption and cost-effective approaches are needed to ensure adequate intake of nutrients among children.

### Contribution of food items to children’s nutrient intakes

Cereal-based foods largely contributed to the intake of nutrients among the study children. Sorghum in the form of porridge, bread and enjera was the children’s primary source of energy, protein, calcium, zinc, iron, thiamin, riboflavin, niacin, and vitamin C during both the lent fasting and non-fasting periods. Tef (*Eragrostis tef)* in the form of gruel, enjera and porridge was also the second most important food sources of nutrient intakes in the study population during both periods. In contrast, in Mexico, different food items such as soups, stews, sweetened bread, dried beans cookies, fruit, tortillas, milk, eggs, and traditional beverages were identified the top food sources of energy and nutrients among infants, toddlers, and young children [47]. Similarly, milk has been reported as the most important source of energy and nutrient intakes among infants and toddlers in Philippines and China [48, 49].

In the study setting, children were served only single food items (sorghum and/or tef) at different times. This is consistent with a study conducted by Motuma et al., [44] who reported that only one food item was processed into various forms and presented to the child at different serving episodes. Therefore, the observed high risk of nutrient inadequacy in the current study population may be attributed to the monotonous, chiefly plant-based diets. These results are largely supported by other dietary survey conducted among rural Zambian children of 4–8 years old [50].

Legumes (such as beans, chickpea, grass pea, peas, vetch, and lentils) were consumed by 58.9 and 49.2% of the infants and young children respectively in the religious fasting and non-fasting periods. As it is prepared in the form of shiro Tsebhi (stew) with very small amounts, its contribution to the children’s nutrient intake was low. The consumption of legumes in small amounts was similar for children of ages 24–48 months in Bangladesh [41]. There is suggestive evidence that complementary foods that are mainly plant-based foods provide insufficient amounts of iron, zinc, and calcium to meet the recommended nutrient intakes for children of 6–23 months of age [29, 51]. Similarly, the principal sources of the above nutrients during both periods were plant source foods such as sorghum and tef in the form of gruel, bread, enjera and porridge across all age groups of the study children. Milk and eggs contributed respectively 18 and 6.6% in lent fasting period and 17.3 and 18.7% in the non-fasting period for the total calcium intake of the study children. In the same way, eggs contributed 12.6% of the total zinc intakes. Surprisingly, sesame, linseed, and enjera which were prepared from the mixture of sorghum and tef were good source of calcium in the study area. Another observation was that enjera and bread which was prepared by mixing of sorghum and maize were rich sources of iron. Thus, to improve the palatability and intake of nutrients among children, homemade/traditional food preparation and processing techniques such as mixing should be promoted [22, 44].

Egg was the most important food sources of vitamin A (71% of the total vitamin A intake) across all age groups of the study children. Only 12.5 and 34.7% of the study children consumed egg in the religious fasting and non-fasting periods, respectively. This indicates that other food items that were more consumed by the infants and young children such as sorghum had (0.0%) vitamin A content. The low consumption of egg among the study group is also comparable with a previous study in rural Tigray [22]. With regard to B-vitamins, cereal foods such as sorghum and wheat for thiamin and sorghum and tef for niacin accounted for more than 50% of their total intakes. This is in line with earlier reports from Ethiopia, which showed that consumption of cereal foods was widespread [19, 52, 53]. Although only 10.1% children consumed dairy products, it contributed to 18.8% of the total riboflavin intakes- the second-highest source of riboflavin next to sorghum among the study children. Mengistu et al., [17] also reported that dairy products were the highest sources of food groups for riboflavin intake among infants and young children. In addition to the presence of low vitamin A source foods, the consumption of other fruits and vegetables in the present study was 13.2%, which is comparable with the national figs [5].; Sorghum (63.8%) was the top source of vitamin C among the study population, contributing to the high prevalence of inadequate vitamin C intakes. These findings highlight the importance of nutrition education and intervention for the study children to improve their dietary structures based on the available resources.

### Strengths and limitations

Data were collected from a representative sample to provide information about the broad assessment of energy and nutrient intakes, and their corresponding food sources among 6–23 months old children of the study area during the lent fasting and non-fasting periods. The collection of data during the lent fasting and non-fasting periods from the individual study population and the use of validated interactive 24-h recall technique to reduce recall bias were some of strengths of the study. However, this study had the following limitations. The study relied on caregiver’s ability to recall accurately on child food intake such as mixed dishes and supplements. Although efforts were made to search the foods that could match the equivalent foods in the national database, it was difficult to estimate the nutrient intakes from a mixed prepared food for those not listed in the national or other food composition database. This could also over or underestimate the nutrient intakes in this population. In such cases, calculation of the nutrient intake was made separately from the available food composition database. Temporal variation was not considered as the differences in dietary intakes in the harvest and lean seasons would influence dietary nutrient intake.

## Conclusions and recommendations

The present study had shown a low dietary diversity and low nutritional quality of foods consumed among the study children during both the lent fasting and non-fasting periods. Also, it was possible to observe a high prevalence of stunting as well as deficits in energy and nutrients among children. Cereals mainly sorghum and tef were the first sources of energy and the top source of other key nutrients across all age groups of the study children. The nutrient-rich foods such as ASFs are almost missing from the diet chiefly during the lent fasting period and it could impact on the dietary diversity and nutritional status of children. Therefore, interventions that improve complementary feeding practices by optimizing the use of locally available foods and strengthening social and behavior change communication are urgently needed.

## Data Availability

The datasets used and/or analyzed during the current study are available from the corresponding author on reasonable request.
